# Stevens–Johnson syndrome/toxic epidermal necrolysis in an orthotopic liver transplant recipient: a case report

**DOI:** 10.1093/jscr/rjad739

**Published:** 2024-01-16

**Authors:** Chun-Sing Huang, Emily Strouphauer, Christine O’Mahony, Nhu T N Galván, Ronald Cotton, John Goss, Abbas Rana

**Affiliations:** Division of Abdominal Transplantation, Michael E. DeBakey Department of General Surgery, Baylor College of Medicine, Houston, TX, United States; School of Medicine, Baylor College of Medicine, 1 Baylor Plaza, Houston, TX, United States; Division of Abdominal Transplantation, Michael E. DeBakey Department of General Surgery, Baylor College of Medicine, Houston, TX, United States; Division of Abdominal Transplantation, Michael E. DeBakey Department of General Surgery, Baylor College of Medicine, Houston, TX, United States; Division of Abdominal Transplantation, Michael E. DeBakey Department of General Surgery, Baylor College of Medicine, Houston, TX, United States; Division of Abdominal Transplantation, Michael E. DeBakey Department of General Surgery, Baylor College of Medicine, Houston, TX, United States; Division of Abdominal Transplantation, Michael E. DeBakey Department of General Surgery, Baylor College of Medicine, Houston, TX, United States

**Keywords:** liver transplantation, Stevens–Johnson syndrome, rare diseases, adverse drug reactions

## Abstract

Stevens–Johnson syndrome/toxic epidermal necrolysis (SJS/TEN) is a rare spectrum of acute, mucocutaneous drug reactions characterized by epidermal necrosis of the skin and mucous membranes with progressive multiorgan failure. Cutaneous presentation of SJS/TEN is similar to that of acute graft-versus-host disease, creating a diagnostic dilemma in solid-organ transplant recipients presenting with diffuse, erythematous eruptions, skin sloughing, and systemic sequelae, reflective of both diseases. This case report details a 48-year-old woman post-orthotopic liver transplantation (OLT) who developed a diffuse, painful, morbilliform rash with progressive desquamation, along with corresponding pathological analysis indicative of SJS/TEN. There are few documented reports of SJS/TEN in solid-organ transplant recipients, and this case illustrates successful intervention and resolution of SJS/TEN in an OLT recipient while managing intraabdominal sepsis and an episode of acute rejection. Despite its rarity, prompt diagnosis of SJS/TEN and the implementation of tailored therapeutic strategies are crucial in the care of solid-organ transplant recipients.

## Introduction

Stevens–Johnson syndrome/toxic epidermal necrolysis (SJS/TEN) is a rare spectrum of acute, mucocutaneous, hypersensitivity reactions characterized by epidermal necrosis of the skin, leading to electrolyte imbalances, infection, multiorgan failure, and death [1]. Initial presentation of SJS/TEN includes nonspecific symptoms, including fever and malaise, followed by widespread, tender erythroderma and rapid progression of blistering rashes, erosions on the face, trunk, and mucosal surfaces, and painful skin sloughing [2]. Although the etiology of SJS/TEN is unknown, it is hypothesized that altered drug metabolism may trigger a T-cell-mediated cytotoxic reaction to drug antigens within keratinocytes, and viral infection may also play a role in pathogenesis [3].

An estimated 1–2 per million people each year are affected with SJS/TEN [4], though the incidence rate is likely much greater in immunocompromised patients [3]. However, there are few, documented reports of SJS/TEN eruptions in solid-organ transplant recipients. After transplantation, patients typically remain on lifelong multimodal, immunosuppressive therapy and prophylactic antibiotic regimens. Several prophylactic agents, such as trimethoprim-sulfamethoxazole, are implicated as causes of SJS/TEN [3]. Diagnosis of SJS/TEN in solid-organ transplant recipients is complicated by its close clinical resemblance to acute graft-versus-host disease (GVHD), creating a diagnostic and therapeutic dilemma. Here, we describe a case of SJS/TEN in a 48-year-old liver transplant recipient one month out from her transplantation, with punch biopsy histopathology illustrating interface dermatitis with vacuolar changes, necrotic keratinocytes, and an inflammatory infiltrate composed of lymphocytes, histiocytes, and scattered eosinophils.

## Case report

We present the case of a 48-year-old woman who developed SJS/TEN following orthotopic liver transplant. She had a routine laparoscopic cholecystectomy in 2002 complicated by bile duct injury. She then underwent hepaticojejunostomy and had recurrent strictures requiring frequent stent placement. She eventually developed secondary biliary cirrhosis, decompensated portal hypertension, and recurrent cholangitis and was listed at our center for liver transplantation. She was otherwise healthy and remaining pre-operative workup was unremarkable.

**Figure 1:**
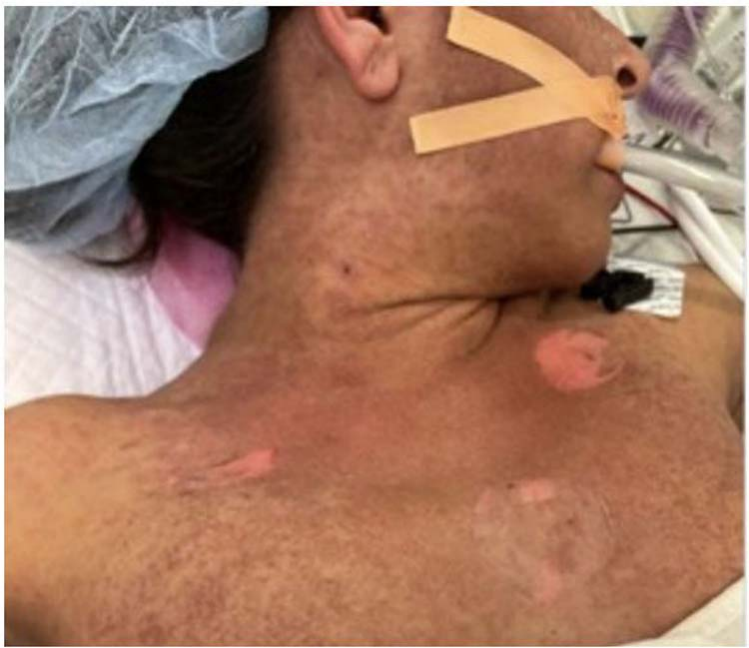
Initial photo of the rash in the operating room during her takeback surgery.

She underwent orthotopic liver transplant on 8 November 2022 from a non-living, unrelated donor. Notably, we discovered a sizable, chronic, partially occlusive portal vein thrombus, promptly removed before the portal anastomosis. Her initial post-operative course was normal, and she was discharged on post-operative day 7. However, she was found to have a new portal vein thrombosis 2 weeks post-transplant, which was managed with endovascular stenting through trans-splenic access. A total of 1 month after transplantation, she developed worsening fevers, and computed tomography (CT) imaging revealed pneumoperitoneum concerning for perforation, so she was taken for exploratory laparotomy. No perforation was identified, but purulence was seen and sent for culture. Notably, during the procedure, we observed the onset of a maculopapular rash and bullae across her chest and torso (Fig. 1), accompanied by persistent fevers reaching 103°F and a newfound pancytopenia. Her white blood cell (WBC) count decreased from 10.1 to 0.3 and her platelet count decreased from 237 to 47.

**Figure 2:**
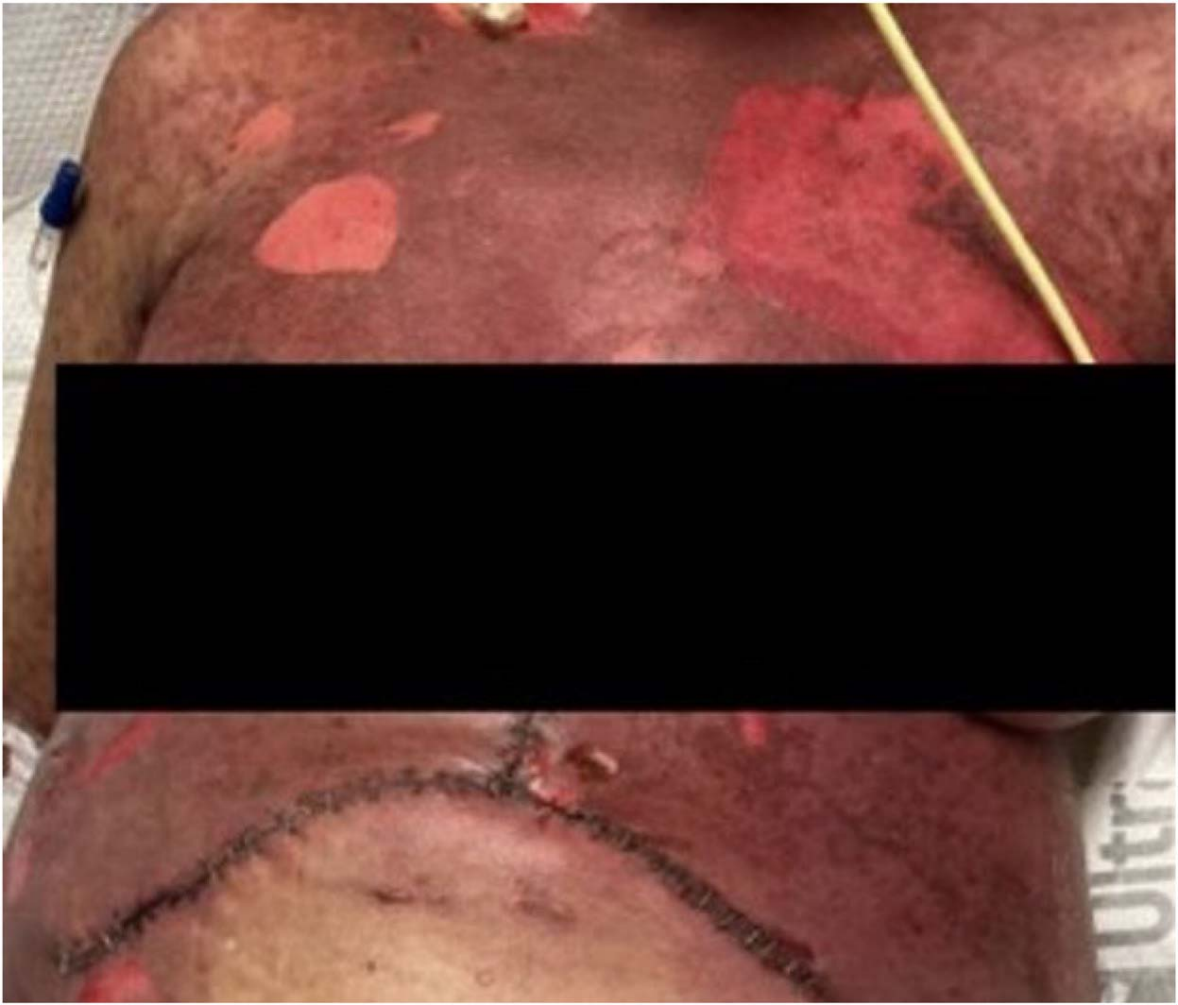
Further progression of her rash a week following developing SJS. She also had significant mucosal sloughing which is not shown in this photo.

We obtained a skin biopsy with concerns about SJS or GVHD, revealing interface dermatitis—a nonspecific finding for SJS, GVHD, and bullous erythema multiforme. HLA testing was negative for GVHD and unlikely given the donor was older and our recipient was relatively young. We treated the leukopenia with filgrastim, a granulocyte colony-stimulating factor, and stopped many of her medications associated with SJS—including switching Bactrim to Pentamidine and Vancomycin to Linezolid. In consultation with hematology and dermatology, we also treated her with steroids, intravenous immunoglobulin (IVIG), and infliximab. Her immunosuppression regimen transitioned from tacrolimus to cyclosporine, aligning with the current literature’s recommendations for treating SJS. We initiated local wound care using iodinated Vaseline gauze, observing the development of extensive bullae on her torso and extremities (Fig. 2), along with gastrointestinal involvement leading to oral mucosal sloughing and diarrhea.

The pancytopenia and skin/mucosal sloughing resolved after 2–3 weeks of the above treatment. Unfortunately, following this therapy, she developed acute rejection that resolved with pulse-dose steroids. She remained hospitalized for 4-months given a persistent intra-abdominal infection. Presently, she has been discharged and continues to do well; her liver graft functions optimally, the portal vein remains unobstructed, she tolerates oral intake, ambulates with ease, and her infections have resolved.

## Discussion

SJS/TEN is a rare diagnosis associated with significant morbidity and mortality. Patients typically present with diffuse, erythematous eruptions, skin sloughing, and systemic sequelae, and diagnosis is confirmed by skin biopsy showing full-thickness dermal necrosis due to extensive keratinocyte apoptosis [5]. Limited cases hinder the establishment of clear SJS/TEN management protocols beyond discontinuing the offending drug, risking sepsis and death with delayed care. This poses a significant concern for solid organ transplant recipients, given that routine post-transplant medications like penicillins and sulfa drugs are known triggers of SJS/TEN [6].

This case of a 48-year-old orthotopic liver transplantation (OLT) recipient, experiencing a painful rash with progressive desquamation, underscores the diagnostic challenges of SJS/TEN in transplant recipients. Considering her recent OLT, clinical presentation, and histologic findings, the differential diagnosis encompassed both SJS/TEN and GVHD. The incidence of acute GVHD after OLT is an estimated 0.1–2% [7], though severe forms may present with bullae, skin sloughing, and pancytopenia, closely resembling the presentation of SJS/TEN as in our patient. Differentiation between SJS/TEN and GVHD is guided by clinical context, HLA typing, and histopathology. Acute GVHD after OLT often presents with diarrhea secondary to cytotoxic T-cell attack on the gastrointestinal mucosa, as well as peripheral blood chimerism [8]. Further, the risk of acute GVHD after OLT increases with increasing recipient age (>65 years) [9], which made this diagnosis less likely in our patient. Though both diseases are partially mediated by cytotoxic T cells against epithelial tissue, recent immunohistologic studies suggest that TEN/SJS may have a greater relative depletion of CD4+ T lymphocytes compared to acute GVHS [10].

Distinguishing between SJS/TEN and acute GVHD is clinically necessary as the approach to treatment differs. First line treatment for acute GVHD for OLT consists of either intravenous methylprednisolone or oral prednisone [11], whereas SJS/TEN treatment is individualized on a case-by-case basis. There are few documented reports of SJS/TEN in solid-organ transplant recipients, and this case illustrates successful intervention and resolution of SJS/TEN with cyclosporine, infliximab, excellent wound care, and most importantly removal of potential instigating medications. Further investigation is necessary to develop a diagnostic algorithm and treatment for SJS/TEN in solid-organ transplant recipients.
